# Plasmodium vivax Malaria Complicated With Acute Respiratory Distress Syndrome: A Case Report

**DOI:** 10.7759/cureus.66189

**Published:** 2024-08-05

**Authors:** Sief Addeen M Khasawneh, Riyadh Hammamy, Afra Elhassan, Mohamed Fawzi Mudarres

**Affiliations:** 1 Internal Medicine, Hamad Medical Corporation, Doha, QAT

**Keywords:** vivax, plasmodium, malaria, falciparum, ards

## Abstract

*Plasmodium vivax *(*P. vivax*) malaria was long considered to have low mortality. Severe malaria was classically associated with *Plasmodium falciparum *(*P. falciparum*). However, there is growing evidence that severe and complicated forms of v*ivax* malaria may be more widespread than previously assumed. We report a case of a 32-year-old female patient with acute respiratory distress syndrome (ARDS) as a complication of *P. vivax* malaria who has recovered completely following starting intravenous antimalarial medications and noninvasive ventilation (NIV).

## Introduction

Malaria is an acute blood infection caused by any of the species of the *Plasmodium* genus. It is mainly caused by *Plasmodium* *falciparum* (*P. falciparum*) and *Plasmodium vivax *(*P. vivax*), with *P. falciparum* causing the largest burden of disease [1]. *P.* *vivax* malaria was long considered to have low mortality. Currently, there is growing evidence that severe and complicated forms of vivax malaria may be more widespread than previously assumed. Acute respiratory distress syndrome (ARDS) is the most important pulmonary manifestation of malaria, with high mortality [2]. Most patients with ARDS require intubation and mechanical ventilation [3].

In this article, we report the diagnosis and the subsequent management of a case of ARDS as a complication of *P. vivax *malaria.

## Case presentation

**Figure 1:**
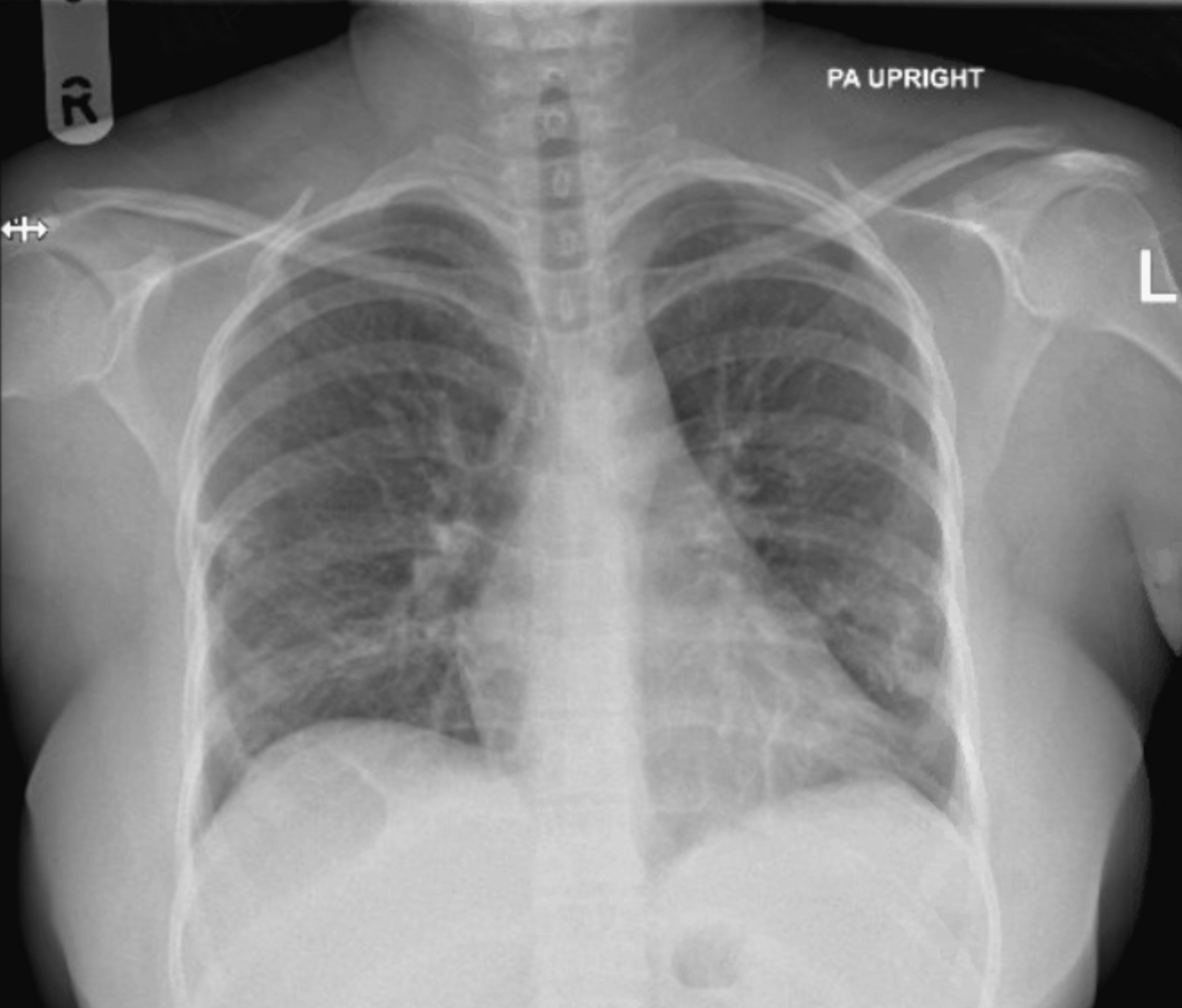
Chest X-ray showing a normal lung parenchyma

A 32-year-old female with no significant past medical history presented with a four-day fever associated with a headache. She resided in a malaria-endemic area of Sudan and had returned from there one month prior to symptom onset. The patient was not on malaria prophylaxis. She experienced a daily fever for one week and had occasional vomiting with no other associated symptoms. Vital signs included a temperature of 39.5 °C, heart rate of 149 bpm, respiratory rate of 20 breaths per minute, blood pressure of 127/79 mmHg, and oxygen saturation (SpO_2_) of 100%. An electrocardiogram (ECG) showed sinus tachycardia. The heart rate normalized after fever control with intravenous paracetamol and 1 L of intravenous fluids. Hemoglobin was low at 9.6 g/dL, and the platelet count was low at 110 x 10^3^/uL. Aspartate aminotransferase (AST) was high at 41 U/L, and alanine transaminase (ALT) was high at 63 U/L. The C-reactive protein (CRP) level was high at 134.2 mg/L. The white blood cell (WBC) count and creatinine levels were normal. Blood cultures obtained at that time showed no growth during the following days. The patient was diagnosed with malaria based on a positive rapid diagnostic test (RDT), which was confirmed to be *P. vivax* on a blood smear with a parasitemia of 0.1%. Repeated tests consistently showed *P. vivax* with no identification of *P. falciparum*. An initial chest X-ray, done routinely in the emergency department without associated respiratory symptoms, was normal (Figure 1). She was admitted to the hospital for observation.

**Figure 2:**
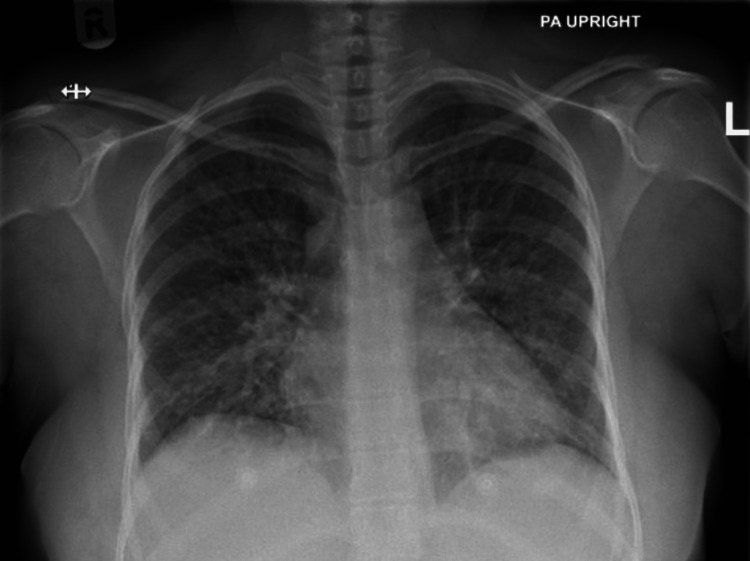
Chest X-ray showing widespread areas of airspace opacities in the bilateral lung zones

**Figure 3:**
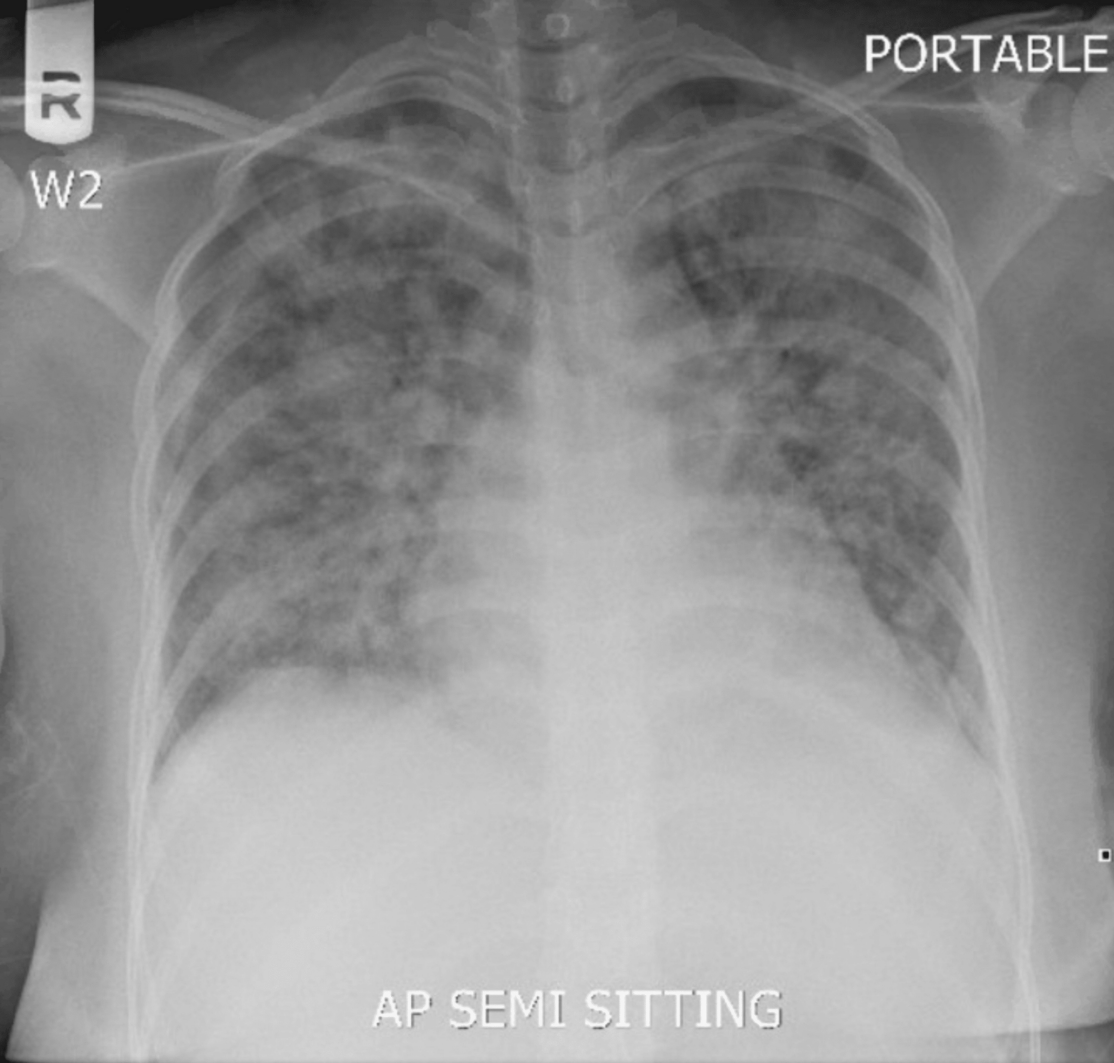
Chest X-ray showing a significant increase in the widespread areas of airspace opacities in the bilateral lung zones, as compared to the previous chest X-ray

The patient received oral chloroquine 1 g. The following day, the patient reported shortness of breath. Vital signs included a temperature of 37.2 °C, heart rate of 120 bpm, respiratory rate of 30 breaths per minute, blood pressure of 131/81 mmHg, and SpO_2_ of 94%. The chest X-ray showed widespread areas of airspace opacities in the bilateral lung zones (Figure 2). The initial arterial blood gases showed a pH of 7.49, pO_2_ of 81 mmHg, pCO_2_ of 27 mmHg, and HCO_3_ of 21.0 mmol/L. Subsequently, intravenous artesunate was started with a dose of 2.4 mg/kg/dose every 12 hours, and oxygen therapy was started using a nasal cannula. However, she had a non-subsiding fever and a progressive increase in her oxygen requirements until she required oxygen therapy via a 10 L non-rebreather mask to maintain her oxygen saturation. Blood tests showed a WBC count of 8.6 x 10^3^/uL, hemoglobin of 8.3 g/dL, platelets of 279 x 10^3^/uL, creatinine of 43 umol/L, ALT of 47 U/L, AST of 55 U/L, N-terminal prohormone of brain natriuretic peptide (NT-pro-BNP) of 100 pg/mL, and CRP of 176.6 mg/L. A repeated chest X-ray (Figure 3), which was performed after two days of the previous X-ray, showed a significant increase in the previously seen bilateral airspace opacities.

Over the three days of admission, the patient had received a total of 3 L of intravenous fluids up to this point as a continuous infusion. Her fluid input and output values were not strictly measured. She did not receive any blood transfusion. Echocardiography showed normal global systolic and diastolic left ventricular function with a biplane left ventricular ejection fraction of 61%. There were no regional wall motion abnormalities. Right ventricular systolic pressure was 57 mmHg (normal value <35 mmHg). Empiric treatment of intravenous Furosemide 20 mg was given as fluid overload, which was a significant differential diagnosis pending the echocardiogram result. However, there was no response.

**Figure 4:**
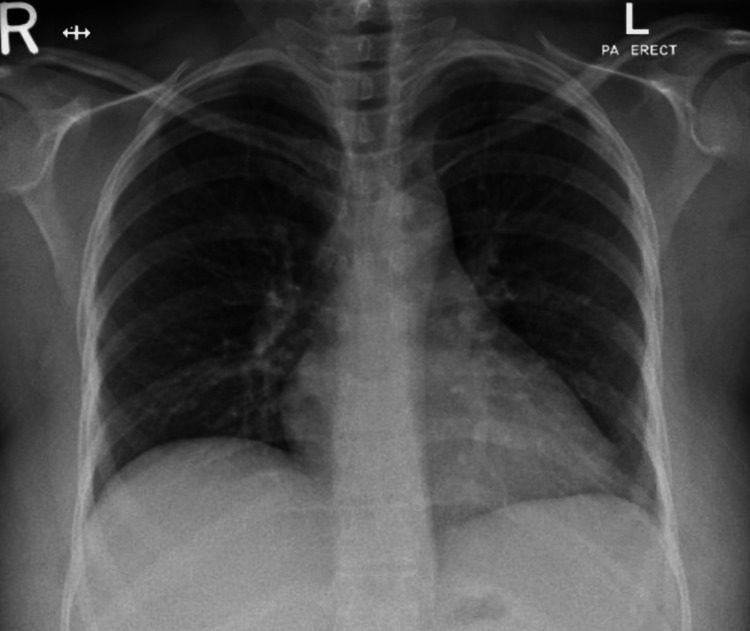
Chest X-ray after treatment showing a significant improvement of the widespread areas of airspace opacities in the bilateral lung zones

The patient was started on noninvasive ventilation (NIV) with inspiratory positive airway pressure (IPAP) of 10 mmHg, expiratory positive airway pressure (EPAP) of 5 mmHg, and FiO_2_ of 0.4. Arterial blood gas (ABG) showed a pO_2_/FiO_2_ ratio of 149, pH of 7.51, pO_2_ of 60 mmHg, pCO_2_ of 32 mmHg, and HCO_3_ of 25.0 mmol/L. The patient started to improve gradually during the following two days. The fever subsided, and the NIV settings were decreased until she was weaned off the NIV and switched to oral artemether-lumefantrine 80 mg/480 mg twice daily for three days. The pO_2_/FiO_2_ chest X-ray showed significant improvement (Figure 4). Oxygen requirements continued improving until they reached room air on the sixth day of admission. She was discharged on primaquine 0.5 mg/kg daily for 14 days.

## Discussion

Malaria is an acute blood infection caused by any of the species of the *Plasmodium* protozoal genus: *P. falciparum, P. vivax, P. malariae, P. ovale, and P. knowlesi* [1]. The World Health Organization (WHO) reported 241 million malaria cases and 627,000 deaths in 2020. *P. falciparum* causes the largest burden of disease, followed by *P. vivax* [4].

The specific group of red blood cells targeted by *Plasmodium* species varies greatly. *P. vivax and P. ovale* only infect immature cells (reticulocytes), which make up just 1%-2% of all red blood cells. On the other hand, *P. falciparum* infects red blood cells indiscriminately, regardless of their age, leading to high levels of parasitemia and potentially severe disease [5].

*P. vivax* is a common source of malaria globally and is the primary cause of this disease in regions outside of Africa. While it was once believed to have a low death rate, recent findings indicate that severe and complicated forms of vivax malaria may be more widespread than previously assumed [6]. In addition, *P. vivax* is known to employ the human Duffy antigen receptor for chemokines (DARC) for red blood cell invasion. However, *P. vivax* has now been observed in Duffy-negative individuals, presenting an increased population at risk and potentially serious public health problems [7].

Increasing parasitemia is classically associated with increasing disease severity. However, according to the WHO malaria guidelines, parasite density thresholds should not be used to determine *vivax* malaria severity [1].

The most important pulmonary complication of malaria is acute lung injury (ALI) and/or ARDS. It has a high mortality rate and is a common feature of severe malaria [2]. The underlying mechanism of ARDS in malaria, primarily researched in the case of *P. falciparum*, is primarily linked to inflammation-induced increased permeability of capillaries or damage to the endothelium, causing widespread damage to the alveoli, even after the parasite is cleared. The extent to which parasite sequestration in the pulmonary microcirculation contributes to the disease is uncertain, as it is a prominent feature of *P. falciparum* but has not been definitively demonstrated in *P. vivax* [8].

ARDS is defined as "the acute onset of respiratory failure, bilateral infiltrates on chest radiograph, and hypoxemia as defined by a pO_2_/FiO_2_ ratio ≤200 mmHg, which is not fully explained by cardiac failure/fluid overload." Patients with ARDS as a complication of malaria should be treated by mechanical ventilation with positive end-expiratory pressure (PEEP), which results in the improvement of ARDS [3]. Our patient was treated with NIV successfully without the need for mechanical ventilation.

Patients with severe malaria should be cared for in a high-dependency or intensive care unit and provided with the necessary hemodynamic support as required. The treatment of choice is intravenous artesunate until the patient is well enough to continue with oral treatment. Close monitoring of fluid balance and status is essential to avoid overfilling, which can worsen the increased permeability of pulmonary capillaries seen in severe malaria. Primaquine should be given after treatment of *P. vivax or P. ovale* for the eradication of hypnozoites [9]. Our patient was treated using the same protocol.

## Conclusions

ARDS can be an important and underrecognized serious complication of *P. vivax* malaria. Due to the limited knowledge regarding this complication, it is necessary to have a high level of suspicion in order to make a diagnosis. NIV can be an appropriate management strategy, and it can be considered before mechanical ventilation. Nevertheless, there needs to be more basic and clinical research to evaluate the burden of this complication as well as to establish proper treatment protocols.
